# Sex differences in hypertension incidence and risk factors: a population-based cohort study in Southern Iran

**DOI:** 10.1186/s12889-024-21082-8

**Published:** 2024-12-23

**Authors:** Abbas Rezaianzadeh, Masoumeh Ghoddusi Johari, Najibullah Baeradeh, Mozhgan Seif, Seyed Vahid Hosseini

**Affiliations:** 1 Department of Community Medicine School of Medicine, Shiraz University of Medical Sciences, Shiraz, Iran; 2 Assistant Professor of Community Medicine, Breast Diseases Research Center, Shiraz University of Medical Sciences, Shiraz, Iran; 3 Department of Public Health, Ferdows Faculty of Medical Sciences, Birjand University of Medical Sciences, Birjand, Iran; 4 Department of Epidemiology, School of Health, Non-Communicable Diseases Research Center, Shiraz University of Medical Sciences, Shiraz, Iran

**Keywords:** Hypertension, Sex, Risk factors, Incidence, Cohort study

## Abstract

**Background:**

Hypertension (HTN) is a major global public health concern. This study aims to identify gender differences to inform more effective prevention strategies and targeted management approaches.

**Methods:**

This prospective cohort study included 7,710 participants aged 40 to 70 years, with a mean follow-up duration of 5.2 years. HTN was defined using European hypertension management guidelines. A Cox regression model was employed to determine factors associated with HTN, adjusting for confounding variables effects.

**Results:**

During the mean follow-up period of 5.2 years, the incidence rate of hypertension was 21.54 per 1,000 person-years, with females exhibiting a higher incidence than males. Several significant predictors of HTN were identified. In men, key risk factors included age (60–70 years, 2.83-fold increase, 95% CI 2.05–3.92), high waist-to-height ratio (5.63-fold increase, 95% CI 2.42–13.07), smoking (2.68-fold increase, 95% CI 1.04–6.91), and opium use (1.93-fold increase, 95% CI 1.06–3.49). In women, significant predictors included age (60–70 years, 3.65-fold increase, 95% CI 2.59–5.15), contraceptive drug use (1.24-fold increase, 95% CI 1.01–1.52), high waist-to-height ratio (1.87-fold increase, 95% CI 1.19–2.92), pre-HTN (3.64-fold increase, 95% CI 3.01–4.40), and kidney stones (1.32-fold increase, 95% CI 1.06–1.65).

**Conclusion:**

This study identified key predictors of hypertension (HTN) with notable gender differences. For men, significant risk factors included age, high waist-to-height ratio, smoking, and opium use; for women, the prominent predictors were age, contraceptive use, pre-HTN, and kidney stones. These findings highlight the need for gender-specific strategies in HTN prevention and management, focusing on modifiable risk factors by gender.

## Introduction

Hypertension (HTN), often referred to as the "silent killer," is one of the most significant risk factors contributing to cardiovascular diseases. Notably, it plays a pivotal role in driving atherosclerosis, which is the primary cause of heart failure and stroke [1, 2]. Unfortunately, it also holds a prominent position as the leading factor behind kidney failure in various nations [3]. This health concern casts a substantial shadow over public health, posing a severe threat to the well-being of society and emerging as a major driver of disability and mortality [2, 4]. Rooted in the intricate interplay of genetic, environmental, and lifestyle factors, hypertension is recognized as a complex condition [5, 6]. HTN, alongside pre-hypertension (pre-HTN), accounts for 8.5 million global deaths due to stroke, ischemic heart disease, other cardiovascular conditions, and kidney disease [7]. It also contributes to 7% of the world's disability-adjusted life years [8]. In the age group of 30 to 79, approximately 1.28 billion adults worldwide grapple with high blood pressure. A significant majority—exactly two-thirds—reside in low- and middle-income countries, underscoring the widespread occurrence of this condition across diverse socioeconomic contexts [9, 10]. However, variations are observed across nations. For instance, within the Eastern Mediterranean region, the prevalence of hypertension is approximately 30% [11]. Similarly, a study focused on Fars province demonstrates that the prevalence of high blood pressure among men and women is 21.44% and 33.53%, respectively [12]. Despite extensive investigations exploring a range of risk factors, including age, sedentary lifestyles, smoking, unhealthy dietary patterns (particularly excessive salt consumption), ethnicity, and alcohol use linked to hypertension, the findings remain contentious [13–16]. These studies often produce conflicting results, further complicated by varying outcomes based on gender [17–20]. Hence, the imperative lies in prevention and strategic management to mitigate the morbidity and mortality associated with hypertension. Since disease management and control differ based on gender, our objective is to investigate gender disparities and differences in the incidence and predictors of hypertension. By doing so, we aim to gain a better understanding of the incidence and risk factors associated with HTN across genders, which can inform preventive strategies and healthcare interventions aimed at improving public health outcomes.

## Methods

The study collected data from the Kharameh cohort, which is part of the Prospective Epidemiological Studies in Iran (PERSIAN) project. The cohort study started in 2014 and includes 18 different areas in Iran, encompassing diverse ethnic groups [21]. Kharameh, a city in the Fars province with a population of 61,580, was included in the Fars Cohort established in 2013.

A total of 10,663 participants aged 40 years and above were enrolled in the study between December 10, 2014, and February 28, 2017. Participants who were diagnosed with hypertension based on specific diagnostic codes were excluded from the analysis. As a result, 7,710 people without hypertension were included in the prospective cohort, and they were followed for an average of five and a half years from study entry until September 2021. Data loss within this cohort was minimal, primarily due to a robust follow-up protocol that included at least seven telephone calls over a two-week period, as well as face-to-face interactions. This comprehensive approach resulted in a high participation rate of 98.7%, which exceeds rates reported in similar cohort studies in Iran [22]. In our study, "time zero" was defined as the date of enrollment. An event was defined as the date of diagnosis, while participants who were not diagnosed by the last follow-up date or who dropped out of the study for any reason were considered censored. Time-to-event was calculated as the duration from time zero to either the date of diagnosis (event) or the date of last follow-up (censoring).

Demographic and lifestyle information was collected through interviews, laboratory experiments, and physical examinations. This included age, gender, marital status, sleep status, fertility history of women, education level, occupation, residence, socioeconomic status, and family history of chronic diseases. Participants' behaviors, such as smoking, alcohol consumption, use of hookah (water pipe), drug use, and physical activity, were also assessed. Fasting blood samples were taken, and measurements of weight and height were obtained to calculate BMI. Laboratory tests measured glucose, triglycerides, HDL, and cholesterol levels. Socioeconomic status was evaluated based on various factors, and average physical activity levels for the past year were determined [23].

### Statistical analysis

Continuous variables were summarized as mean ± standard deviation (SD) if they had a normal distribution, and as median (interquartile range, Q1–Q3) if they did not. Categorical variables were presented as counts and percentages. To examine relationships between variables, Chi-square tests were used for categorical data, while independent t-tests were employed to compare means of continuous variables across groups. Cox regression analysis was used to identify and predict factors associated with HTN.

For the final model, variables that had a *p*-value of less than 0.2 from the univariate analysis were included, along with other clinically relevant factors identified through a literature review. The following confounding variables were adjusted for, along with the significant variables in the final model: Sociodemographic factors: education level, place of residence, employment status, and socioeconomic status. Lifestyle factors: physical activity, hookah use, and alcohol consumption. Medical history: family history of diabetes, existing chronic conditions (e.g., diabetes, stroke, fatty liver, gallstones), and contraceptive use. Clinical measures and medication use: HDL levels, and the use of aspirin and statins. All statistical analyses were conducted using SPSS version 23 and Stata version 12, with data visualization performed using GraphPad Prism version 8. Two-sided *p*-values with a threshold of 0.05 were used to determine statistical significance.

## Result

**Fig. 1:**
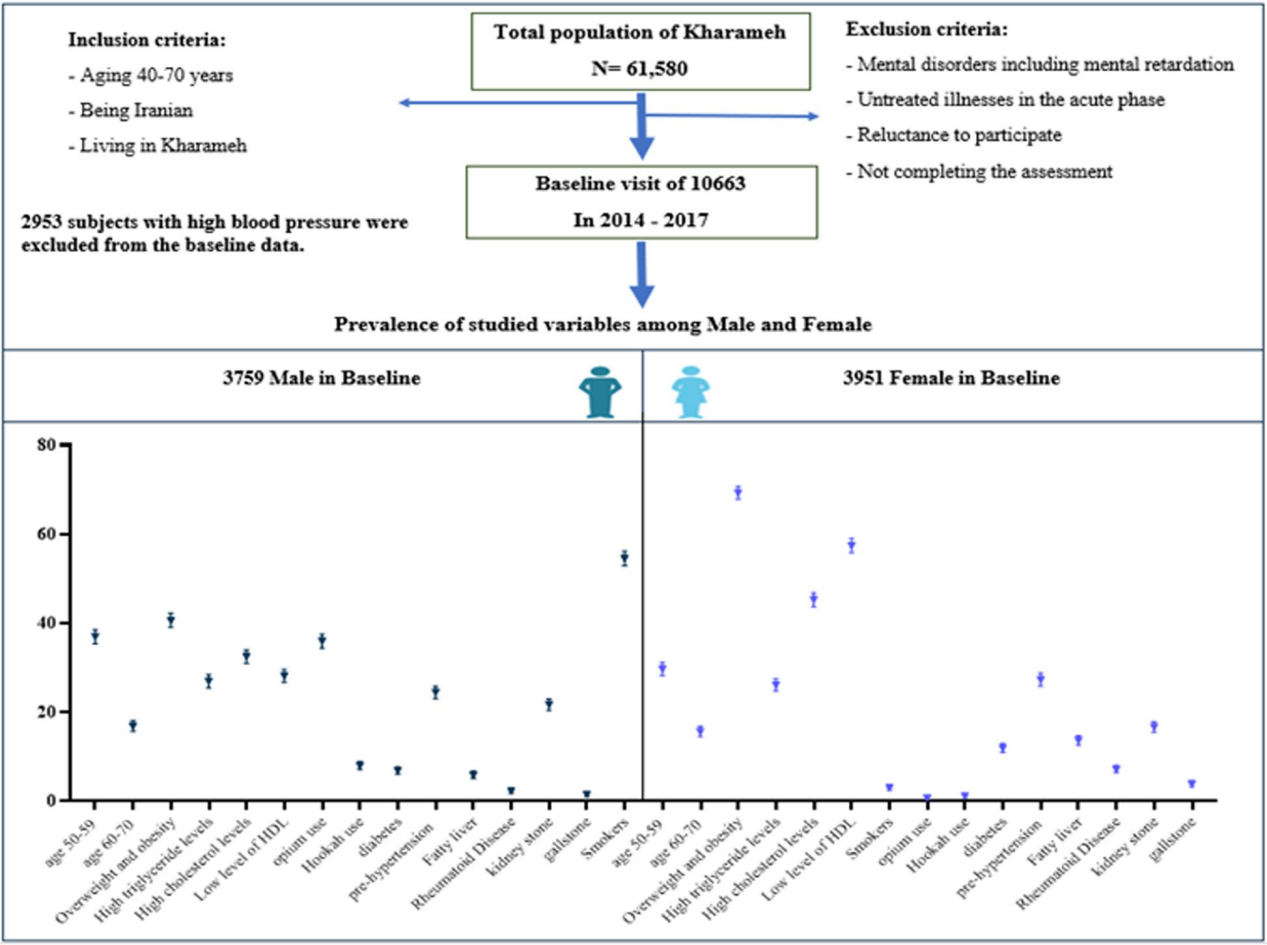
Flow chart for the enrollment process of study and Prevalence of studied variables among male and female in baseline

**Fig. 2:**
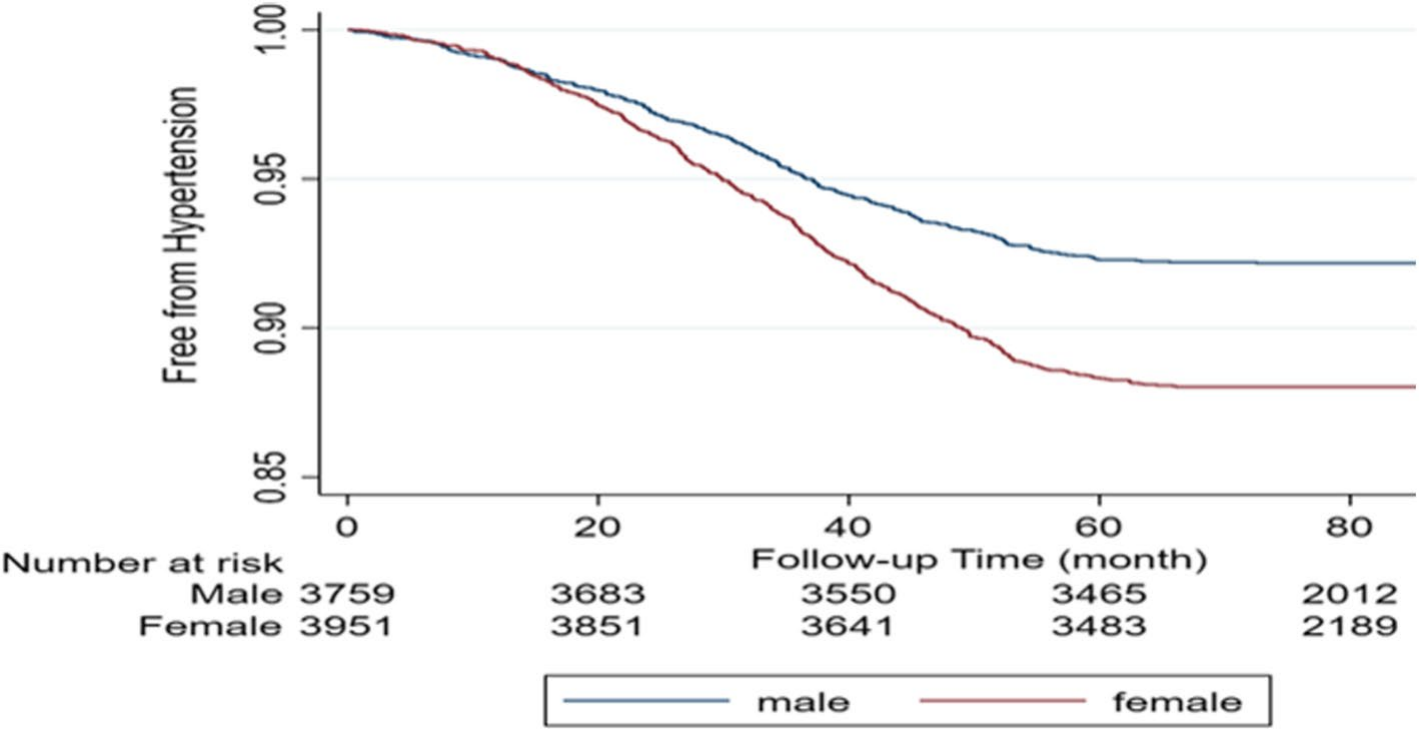
Kaplan–Meier curves for HTN-free survival by gender. (The Kaplan–Meier survival curve demonstrates stabilization beyond the 60-month time point, despite a significant reduction in the number of participants at risk. This pattern can be explained by the study design, in which participants were enrolled over a two-year period. Consequently, some participants had follow-up durations shorter than 60 months and were censored at the end of their respective observation periods. The decrease in the number at risk after 60 months is thus a result of censoring, rather than an increase in event occurrences. The stabilization of the survival curve during this time frame likely reflects a low incidence of events among the remaining cohort)

Table 1 and Fig. 1 compare the characteristics of male and female participants across various demographic and health variables. Significant gender differences were observed in age, BMI, marital status, education level, place of residence, employment status, and the prevalence of chronic diseases such as hypertension, diabetes, stroke, and fatty liver (detailed statistics in Table 3). Notably, women were more likely to be overweight or obese, illiterate, residing in rural areas, and unemployed. Additionally, women had higher rates of diabetes, pre-hypertension, and fatty liver disease than men. In contrast, men had higher rates of smoking (54.56%), opium use (36.02%), and alcohol consumption (8.67%). Furthermore, women reported a greater use of steroids (2.94%), non-steroidal anti-inflammatory drugs (11.95%), and statins (5.92%). Figure 2 shows Kaplan–Meier survival curves for hypertension-free survival, separated by gender. The curves show a significant difference in hypertension-free survival rates between male and female participants, with males having a higher rate of survival free from hypertension (log-rank *P* < 0.001).

**Table 1:** Demographic, lifestyle, clinical and behavioral variables of the participants according to the sex status

Demographic and lifestyle variables of the participants						The clinical and behavioral variables of the participants				
Characteristics			Male	Female	*P*-value	Characteristics		Male	Female	*P*-value
Age (Years)		40-49	1733 (46.1)	2160 (54.67)	<0.001	Blood pressure	Hypotension	247 (6.57)	344 (8.71)	<0.001
		50-59	1392 (37.03)	1173 (29.69)			Normotensive	2592 (68.95)	2528 (63.98)	
		60-70	634 (16.87)	618 (15.64)			Pre- HTN	920 (24.47)	1079 (27.31)	
BMI (kg/m^2^)		<18.4	287 (7.64)	79 (2)	<0.001	Diabetes	No	3502 (93.16)	3480 (88.08)	<0.001
		18.5-24.9	1942 (51.7)	1131 (28.65)			Yes	257 (6.84)	471 (11.92)	
		25-29.9	1282 (34.13)	1815 (45.97)		Stroke	No	3727 (99.15)	3925 (99.34)	0.32
		>30	245 (6.52)	923 (23.38)			Yes	32 (0.85)	26 (0.66)	
Marital status		Unmarried	25 (0.67)	147 (3.72)	<0.001	Fatty Liver	No	3537 (94.09)	3412 (86.36)	<0.001
		Widowed or divorced	21 (0.56)	482 (12.2)			Yes	222 (5.91)	539 (13.64)	
		Married	3713 (98.78)	3322 (84.08)		Gallstone	No	3703 (98.51)	3798 (96.13)	<0.001
Education level		Illiterate	1382 (36.77)	2290 (57.96)	<0.001		Yes	56 (1.49)	153 (3.87)	
		Diploma and below	2056 (54.7)	1521 (38.5)		Kidney stone	No	2942 (78.27)	3292 (83.32)	<0.001
		Academic	321 (8.54)	140 (3.54)			Yes	817 (21.73)	659 (16.68)	
Living place		Urban	1635 (43.52)	1522 (38.54)	<0.001	Rheumatoid disease	No	3673 (97.71)	3666 (92.79)	<0.001
		Rural	2122 (56.48)	2427 (61.46)			Yes	86 (2.29)	285 (7.21)	
Employed		No	459 (12.21)	2801 (70.89)	<0.001	History of swelling in the body, especially the legs	No	3461 (92.07)	3146 (79.63)	<0.001
		Yes	3.300 (87.79)	1150 (29.11)			Yes	298 (7.93)	805 (20.37)	
Physical activity		Light	1.001 (26.64)	693 (17.55)	<0.001	Urinary problems	No	1999 (53.18)	1845 (46.7)	<0.001
		Moderate	617 (14.42)	1276 (32.31)			Yes	1760 (46.82)	2106 (53.3)	
		High	570 (15.17)	1372 (34.74)		Gastroesophageal reflux disease (GERD)	No	3040 (80.87)	3139 (79.45)	0.11
		Severe	1569 (41.76)	608 (15.4)			Yes	719 (19.13)	812 (20.55)	
Wealth score index		Low income	1704 (45.33)	2384 (60.34)	<0.001	History of hit on head with anesthesia	No	3446 (91.67)	3765 (95.29)	<0.001
		Low- middle income	946 (25.17)	818 (20.7)			Yes	313 (8.33)	186 (4.71)	
		Middle-high income	1016 (27.03)	704 (17.82)		Recurrent headache attacks	No	3178 (84.54)	2685 (67.96)	<0.001
		High income	93 (2.47)	45 (1.14)			Yes	581 (15.46)	1266 (32.04)	
Unintentional naps		No	1931 (51.37)	1966 (49.76)	0.15	Dizziness attacks	No	3540 (94.17)	3332 (84.33)	<0.001
		Yes	1828 (48.63)	1985 (50.24)			Yes	219 (5.83)	619 (15.67)	
Use of sleeping pills		No	3558 (94.65)	3624 (91.72)	<0.001	Tinnitus attack	No	3518 (93.59)	3580 (90.61)	<0.001
		Yes	201 (5.35)	327 (8.28)			Yes	241 (6.41)	371 (9.39)	
Use of infertility drug		NO	Na	3603 (94.92)		Osteoporosis	No	3693 (98.24)	3465 (87.7)	<0.001
		YES		193 (5.08)			Yes	66 (1.76)	486 (12.3)	
Use of contraceptive drug		NO	Na	1259 (31.87)		Chronic Back Pain	No	3031 (80.63)	2743 (69.43)	<0.001
		YES		2691 (68.13)			Yes	728 (19.37)	1208 (30.57)	
Infertile women		NO	Na	3565 (93.2)		Joint pain	No	3015 (80.21)	2473 (62.59)	<0.001
		YES		262 (6.8)			Yes	744 (19.79)	1478 (37.41)	
Menopausal		NO	Na	2216 (56.1)		High cholesterol level	No	2532 (67.43)	2160 (54.68)	<0.001
		YES		1735 (43.9)			Yes	1223 (32.57)	1790 (45.32)	
Hair loss		NO	1565 (41.63)	1566 (39.64)	0.074	High triglyceride level	No	2725 (72.57)	2916 (73.82)	0.21
		YES	2194 (58.37)	2385 (60.36)0			Yes	1013 (27.43)	1034 (26.18)	
Green iris color		NO	3335 (88.72)	3657 (92.56)	<0.001	HDL (mg/dl)	<40 for males and <50 for females	1059 (28.26)	2269 (57.59)	<0.001
		YES	424 (11.28)	294 (7.44)			≥40 for males and ≥50 for females	2689 (71.74)	1671 (42.41)	
Mobile phone usage		NO	221 (5.88)	1.118 (28.3)	<0.001	Opium use	No	2405 (63.98)	3922 (99.27)	<0.001
		YES	3538 (94.12)	2833 (71.7)			Yes	1354 (36.02)	29 (0.73)	
Tubectomy		NO	Na	2055 (52.01)		Hookah use	No	3458 (91.99)	3904 (98.81)	<0.001
		YES		1896 (47.99)			Yes	301 (8.01)	47 (1.19)	
Family history of diabetes	First degree	No	2616 (69.59)	2450 (62.01)	<0.001	Smoking	No	1708 (45.44)	3830 (96.94)	<0.001
		Yes	1143 (30.41)	1501 (37.99)			Yes	2051 (54.56)	121 (3.06)	
	Second degree	No	3162 (84.12)	3041 (76.97)	<0.001	Alcohol consumption	No	3433 (91.33)	3951 (100)	<0.001
		Yes	597 (15.88)	910 (23.03)			Yes	326 (8.67)	0 (0.00)	
Family history of HTN	First degree	No	2115 (56.26)	1914 (48.44)	<0.001	Aspirin	No	3567 (94.89)	3804 (95.6)	0.003
		Yes	1644 (43.74)	2037 (51.56)			Yes	192 (5.11)	147 (3.72)	
	Second degree	No	3272 (87.04)	3176 (80.38)	<0.001	Non-steroidal drugs	No	3584 (95.34)	3479 (88.05)	<0.001
		Yes	487 (12.96)	775 (19.62)			Yes	175 (4.66)	472 (11.95)	
Family history of stroke	First degree	No	3270 (86.99)	3400 (86.05)	0.22	Statin	No	3661 (97.39)	3717 (94.08)	<0.001
		Yes	489 (13.01)	551 (13.95)			Yes	98 (2.61)	234 (5.92)	
	Second degree	No	3549 (94.41)	3695 (93.52)	0.10	Steroid	No	3708 (98.64)	3835 (97.06)	<0.001
		Yes	210 (5.59)	256 (6.48)			Yes	51 (1.36)	116 (2.94)	
						Immunosuppressive agents	No	3750 (99.76)	3923 (99.29)	0.003
							Yes	9 (0.24)	28 (0.71)

### The crude and age-standardized incidence density rates of hypertension by sex

Our results show that the median follow-up time was 64.5 months (IQR: 14.03 months), and the median survival time was 32.5 months (IQR: 22.3 months). The age-standardized incidence density rate of hypertension (HTN) was 21.54 per 1,000 person-years (95% CI: 20.1–23.36). Stratified by sex, the rate was 16.06 per 1,000 person-years (95% CI: 14.24–17.88) among men, compared to 27.37 per 1,000 person-years (95% CI: 24.82–29.93) among women.

### Predictors of incident hypertension based on sex

**Fig. 3:**
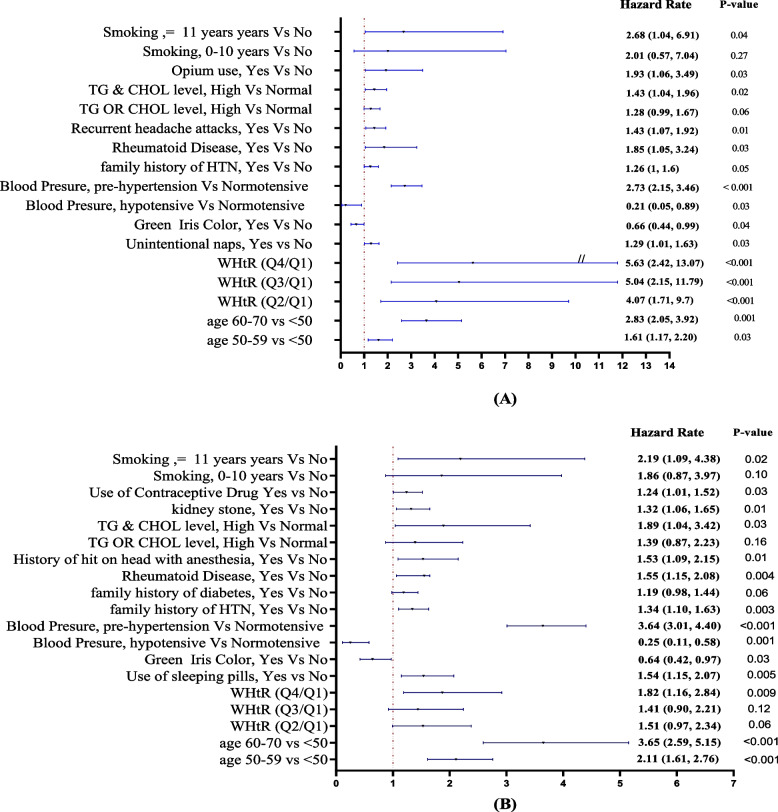
**A** predictors of incident hypertension in male. **B** predictors of incident hypertension in female

**Fig. 4:**
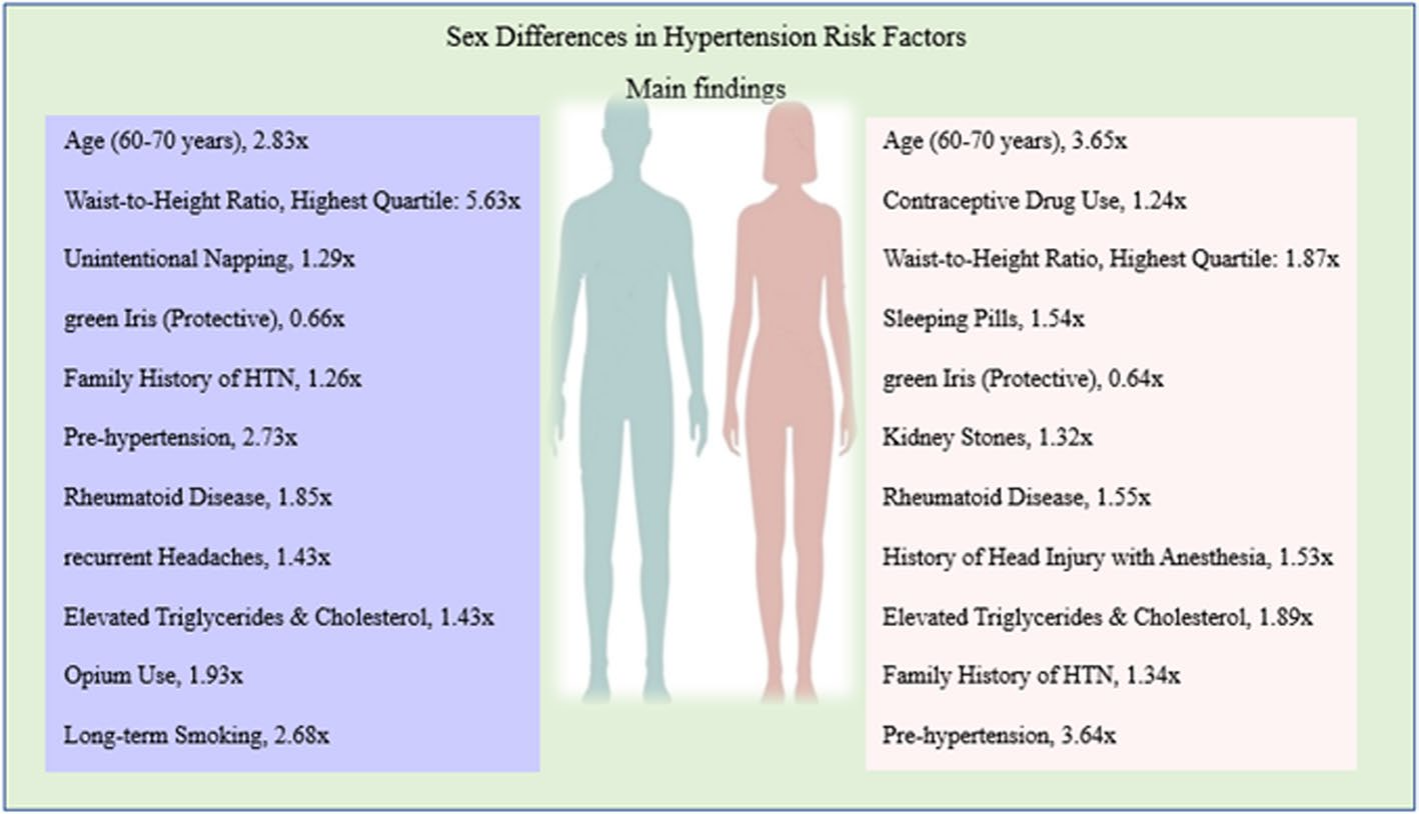
Risk factors of hypertension by sex

Due to the distinct baseline variables observed between the sexes (as outlined in Table 1 and Figs. 2 and 3), we conducted separate univariate and multivariate analyses for each gender. After the bivariate analysis, we employed a multivariable Cox regression model to control for confounding factors within each sex (Fig. 4).

### Findings in the male population

Our study identified several significant associations between various factors and the hazard rate (HR) of hypertension (HTN) in men. Men aged 60–70 years had a 2.83-fold higher HR for HTN compared to those aged 40–50 years (95% CI: 2.05–3.92, *P* < 0.05). Men in the highest quartile of waist-to-height ratio had a 5.63-fold higher HR than those in the lowest quartile (95% CI: 2.42–13.07, *P* < 0.05). Certain lifestyle factors were also linked to HTN risk. Men who unintentionally napped had a 1.29-fold higher HR (95% CI: 1.01–1.63, *P* < 0.05), while those with green irises had a 34% lower HR than men with other iris colors (95% CI: 0.44–0.99, *P* < 0.05). A family history of HTN in first-degree relatives was associated with a 1.26-fold higher risk (95% CI: 1.01–1.6, *P* < 0.05). Men with pre-HTN had a 2.73-fold higher HR than normotensive individuals (95% CI: 2.15–3.46, *P* < 0.05). Rheumatoid disease was linked to a 1.85-fold higher HR (95% CI: 1.05–3.24, *P* < 0.05). Recurrent headaches (HR: 1.43, 95% CI: 1.07–1.92, *P* < 0.05) and elevated triglyceride and cholesterol levels (HR: 1.43, 95% CI: 1.04–1.96, *P* < 0.05) were also associated with higher HTN risk. Opium use was associated with a 1.93-fold higher HR than non-users (95% CI: 1.06–3.49, *P* < 0.05). Long-term smokers (over 11 years of smoking) had a 2.68-fold higher HR than non-smokers (95% CI: 1.04–6.91, *P* < 0.05). Additionally, moderate interactions between opium use and HDL levels, as well as between smoking and opium use, were observed in the univariate analysis of hypertension (HTN) risk among men.

### Findings in the female population

Our multiple Cox regression analysis identified several significant predictors of hypertension (HTN) in women. Women aged 60–70 years had a 3.65-fold higher HR for developing HTN compared to those aged 40–50 years (95% CI: 2.59–5.15, *P* < 0.05). The use of contraceptive drugs was associated with a 1.24-fold increase in HR for HTN (95% CI: 1.01–1.52, *P* < 0.05). Anthropometric measures also demonstrated strong associations with HTN. Women in the highest quartile of waist-to-height ratio had a 1.87-fold higher HR (95% CI: 1.19–2.92, *P* < 0.05) compared to those in the lowest quartile. Lifestyle factors also contributed to HTN risk. Women who used sleeping pills had a 1.54-fold higher HR (95% CI: 1.15–2.07, *P* < 0.05). In contrast, women with green irises had a 0.64-fold lower HR (95% CI: 0.42–0.97, *P* < 0.05) compared to those with other iris colors. A family history of HTN in first-degree relatives was associated with a 1.34-fold increase in risk (95% CI: 1.10–1.63, *P* < 0.05). Several health conditions were associated with elevated HTN risk. Women with pre-HTN had a 3.64-fold higher HR than normotensive individuals (95% CI: 3.01–4.40, *P* < 0.05). Additionally, women with kidney stones had a 1.32-fold higher HR (95% CI: 1.06–1.65, *P* < 0.05), and those with rheumatoid disease exhibited a 1.55-fold increase in risk (95% CI: 1.15–2.08, *P* < 0.05). A history of head injury with anesthesia was linked to a 1.53-fold higher HR (95% CI: 1.09–2.15, *P* < 0.05). Biochemical factors also showed significant associations with HTN. Women with elevated triglycerides (TG) and cholesterol (CHOL) had a 1.89-fold higher HR for HTN (95% CI: 1.04–3.42, *P* < 0.05). Smoking also increased HTN risk, with women who smoked for more than 11 years exhibiting a 2.19-fold higher HR than non-smokers (95% CI: 1.09–4.38, *P* < 0.05). In summary, these findings highlight several key factors associated with HTN risk in women, including age, contraceptive drug use, anthropometric measures, health conditions, and lifestyle choices. Additionally, in the univariate analysis, interactions between triglyceride and cholesterol levels and the waist-to-height ratio were observed (see Tables 2 and 3).

**Table 2:** Demographic and lifestyle predictors of hypertension by sex

Characteristics			Male		Female	
			Crude HR (95% CI)	Adjusted HR (95% CI)	Crude HR (95% CI)	Adjusted HR (95% CI)
Age (Years)		40–49	1	1	1	1
		50–59	2.89 (2.10, 3.99)	1.61 (1.17, 2.20)	2.93 (2.32, 3.70)	2.11 (1.61, 2.76)
		60–70	6.39 (4.63, 8.82)	2.83 (2.05, 3.92)	5.96 (4.72, 7.53)	3.65 (2.59, 5.15)
Waist to height ratio		Q1	1	1	1	1
		Q2	2.15 (1.36, 3.4)	4.07 (1.71, 9.7)	1.24 (0.92, 1.66)	1.53 (0.99, 2.38)
		Q3	3.45 (2.24, 5.31)	5.04 (2.15, 11.79)	1.45 (1.09, 1.93)	1.44 (0.92, 2.24)
		Q4	4.69 (3.09, 7.12)	5.63 (2.42, 13.07)	2.26 (1.74, 2.94)	1.87 (1.19, 2.92)
Education level		Illiterate	1		1	
		Diploma and below	0.59 (0.46, 0.74)		0.48 (0.39, 0.59)	
		Academic	0.58 (0.36, 0.93)		0.31 (0.14, 0.66)	
Living place		Urban	1		1	
		Rural	1.03 (0.82, 1.30)		1.35 (1.11, 1.64)	
Employed		No	1		1	
		Yes	0.78 (0.56, 1.07)		0.77 (0.63, 0.95)	
Physical activity		Light	1		1	
		Moderate	0.66 (0.45, 0.96)		1.14 (0.88, 1.46)	
		High	0.87 (0.611, 1.25)		0.72 (0.55, 0.94)	
		Severe	0.86 (0.65, 1.13)		0.89 (0.65, 1.23)	
wealth score index		Low income	1		1	
		Low- middle income	1.05 (0.80, 1.38)		0.79 (0.62, 1.01)	
		Middle-high income	0.81 (0.61, 1.08)		0.68 (0.51, 0.88)	
		High income	0.38 (0.12, 1.21)		0.65 (0.24, 1.75)	
Unintentional naps		No	1	1	1	
		Yes	1.48 (1.18, 1.87)	1.29 (1.01, 1.63)	1.00 (0.84, 1.20)	
Use of sleeping pills		No	1		1	1
		Yes	0.82 (0.47, 1.43)		1.37 (1.02, 1.83)	1.54 (1.15, 2.07)
Use of infertility drug		NO			1	
		YES			0.48 (0.27,0.85)	
Use of contraceptive drug		NO			1	1
		YES			1.13 (0.93, 1.38)	1.24 (1.01, 1.52)
Infertile women		NO			1	
		YES			0.62 (0.4, 0.97)	
Menopausal		NO			1	
		YES			1.93 (1.60, 2.32)	
Hair loss		NO	1		1	
		YES	1.09 (0.86, 1.38)		1.33 (1.09, 1.61)	
Green iris color		NO	1	1	1	1
		YES	0.78 (0.53, 1.16)	0.66 (0.44, 0.99)	0.64 (0.43, 0.97)	0.64 (0.42, 0.97)
Mobile phone usage		NO	1		1	
		YES	0.64 (0.43, 0.97)		0.74 (0.61, 0.90)	
Tubectomy		NO			1	
		YES			1.20 (1.03, 1.44)	
Family history of diabetes	First degree	No	1		1	1
		Yes	1.06 (0.82, 1.35)		1.27 (1.06, 1.52)	1.19 (0.98, 1.44)
	Second degree	No	1		1	
		Yes	1.04 (0.76, 1.41)		0.88 (0.70, 1.10)	
Family history of HTN	First degree	No	1	1	1	1
		Yes	1.19 (0.95, 1.50)	1.26 (1.01, 1.6)	1.32 (1.10, 1.59)	1.34 (1.10, 1.63)
	Second degree	No	1		1	
		Yes	1.11 (0.80, 1.54)		0.82 (0.64, 1.04)	
Family history of stroke	First degree	No	1		1	
		Yes	0.99, 0.70, 1.39)		1.26 (0.99, 1.60)	
	Second degree	No	1		1	
		Yes	0.77 (0.44, 1.35)		0.80 (0.53, 1.20)	
Marital status	Unmarried		1		1	
	Widowed or divorced		-		3.5 (1.72, 7.34)	
	Married		1.94 (0.27, 13.86)		2.16 (1.07, 4.36)

**Table 3:** Clinical and behavioral predictors of hypertension by sex

Characteristics		Male		Female	
		Crude HR (95% CI)	Adjusted HR (95% CI)	Crude HR (95% CI)	Adjusted HR (95% CI)
Blood pressure	Normotensive	1	1	1	1
	Hypotension	0.15 (0.03,0.62)	0.21 (0.05, 0.89)	0.23 (0.10, 0.52)	0.25 (0.11, 0.58)
	Pre- HTN	3.58 (2.84, 4.51)	2.73 (2.15, 3.46)	4.05 (3.36, 4.88)	3.64 (3.01, 4.40)
Diabetes	No	1		1	
	Yes	1.76 (1.22, 2.53)		1.27 (0.98, 1.64)	
Stroke	No	1		1	
	Yes	1.64 (0.61, 4.39)		2.50 (1.18, 5.28)	
Fatty liver	No	1		1	
	Yes	1.51 (1.008, 2.28)		1.05 (0.81, 1.36)	
Gallstone	No	1		1	
	Yes	3.04 (1.71, 5.43)		1.16 (0.75, 1.80)	
Kidney stone	No	1		1	1
	Yes	1.43 (1.11, 1.84)		1.49 (1.20, 1.85)	1.32 (1.06, 1.65)
Rheumatoid disease	No	1	1	1	1
	Yes	2.04 (1.17, 3.55)	1.85 (1.05, 3.24)	1.56 (1.16, 2.09)	1.55 (1.15, 2.08)
History of swelling in the body, especially the legs	No	1		1	
	Yes	1.64 (1.15, 2.32)		1.39 (1.13, 1.70)	
urinary problems	No	1		1	
	Yes	1.34 (1.06, 1.69)		1.24 (1.03, 1.48)	
Gastroesophageal reflux disease (GERD)	No	1		1	
	Yes	1.17 (0.88, 1.54)		1.25 (1.01, 1.54)	
History of hit on head with anesthesia	No	1		1	1
	Yes	1.32 (0.911, 1.91)		1.81 (1.29, 2.53)	1.53 (1.09, 2.15)
Recurrent headache attacks	No	1	1	1	
	Yes	1.35 (1.01, 1.80)	1.43 (1.07, 1.92)	1.20 (1.00, 1.45)	
Dizziness attacks	No	1		1	
	Yes	1.52 (1.01, 2.30)		1.17 (0.93, 1.49)	
tinnitus attack	No	1		1	
	Yes	1.69 (1.16, 2.46)		1.33 (1.01, 1.76)	
Osteoporosis	No	1		1	
	Yes	0.97 (0.40, 2.36)		1.54 (1.21, 1.95)	
Chronic back pain	No	1		1	
	Yes	1.17 (0.89, 1.54)		1.36 (1.13, 1.64)	
Joint pain	No	1		1	
	Yes	1.12 (0.85, 1.48)		1.38 (1.15, 1.65)	
High cholesterol & triglyceride levels	NO	1	1	1	1
	TG or CHO	1.68 (1.30,2.18)	1.28 (0.99, 1.67)	1.31 (1.07,1.61)	1.39 (0.87, 2.23)
	TG and CHO	1.98 (1.46, 2.69)	1.43 (1.04, 1.96)	1.64 (1.28, 2.08)	1.89 (1.04, 3.42)
HDL	< 40 for males and < 50 for females	1		1	
	≥ 40 for males and ≥ 50 for females	1.18 (0.90, 1.53)		1.04 (0.87, 1.25)	
Opium use	No	1	1	1	
	Yes	0.77 (0.60, 0.99)	1.93 (1.06, 3.49)	0.53 (0.13, 2.16)	
Hookah use	No	1		1	
	Yes	0.78 (0.49, 1.24)		1.27 (0.60, 2.68)	
Smoking	No	1	1	1	1
	0–10 years	0.54 (0.41, 0.71)	2.01 (0.57, 7.04)	1.37 (0.68,2.75)	1.86 (0.87, 3.97)
	≥ 11 years	1.03 (0.76,1.39)	2.68 (1.04, 6.91)	1.79 (1.05, 3.05)	2.19 (1.09, 4.38)
Interaction smoke & opium use		1			
		1.09 (0.58, 2.08)			
		0.49 (0.27, 0.88)			
Interaction opium & HDL		1			
		0.58 (0.34, 1.0)			
Interaction WHR & high cholesterol & triglyceride levels				1	
				0.43 (0.22, 0.82)	
				0.58 (0.33, 1.02)	
				0.53 (0.29, 0.96)	
				0.63 (0.29,1.37)	
				0.30 (0.14, 0.63)	
Alcohol consumption	No	1		NO User	
	Yes	0.55 (0.33, 0.93)			
Creatinine levels		1.92 (1.04, 3.54)		1.86 (1.01, 3.44)	
Aspirin	No	1		1	
	Yes	1.70 (1.12, 2.58)		1.45 (0.97,2.17)	
Non-steroidal drugs	No	1		1	
	Yes	1.50 (0.95, 2.37)		1.62 (1.27, 2.04)	
Statin	No	1		1	
	Yes	2.24 (1.36, 3.72)		1.42 (1.02, 1.98)	
Steroid	No	1		1	
	Yes	1.27 (0.52, 3.08)		0.94 (0.54, 1.64)	
Immunosuppressive agents	No	1		1	
	Yes	1.39 (0.19, 9.93)		0.52 (0.13, 2.11)	

## Discussion

This study offers a comprehensive exploration of sex-specific differences in the incidence and predictors of hypertension (HTN), providing valuable insights into how HTN manifests in men and women. Our findings reveal distinct patterns in risk factors and incidence rates, emphasizing the importance of sex-specific approaches to HTN management.

### Incidence of hypertension based on sex

Our study revealed a higher incidence of HTN in women than in men, with cumulative incidence rates of 13.78% for women and 8.5% for men. The incidence density was similarly higher in women (27.37 per 1000 person-years) compared to men (16.06 per 1000 person-years). These results are consistent with studies suggesting that postmenopausal women face a greater prevalence of HTN due to hormonal changes and increased risk factors, such as hysterectomy or oophorectomy [17, 24, 25]. Conversely, some studies have reported a higher incidence of HTN in men [17, 18]. The elevated incidence of HTN in women observed in our study may be attributed to the age distribution of our female participants, many of whom were postmenopausal and had undergone tubectomy, hysterectomy, or oophorectomy. These procedures are associated with an increased risk of cardiovascular, hypertensive, and metabolic diseases, primarily due to hormonal and metabolic changes, as shown in previous research [26–29].

### Predictors of incident hypertension based on sex

Age emerged as a significant predictor of HTN in both men and women, with individuals aged 60–70 years exhibiting notably higher HR compared to younger participants. This is consistent with the well-established association between increasing age and HTN, mainly due to vascular changes and arterial stiffness [30–32]. Additionally, waist-to-height ratio (WHtR) was a strong predictor in both sexes, with participants in the highest quartile of WHtR displaying significantly higher HR than those in the lowest quartile. WHtR has gained recognition as a superior predictor of cardiovascular risk compared to body mass index (BMI), as it more accurately reflects central obesity, a key factor in HTN [33–38]. Our findings also support that elevated levels of triglycerides and cholesterol are significant risk factors for HTN across both sexes. Individuals with concurrently elevated cholesterol and triglyceride levels were at higher risk of developing HTN compared to those with elevated levels of only one of these factors. This finding is consistent with earlier studies linking high levels of these lipids to HTN [12, 31, 39–41]. However, discrepancies exist in the literature, with some cross-sectional studies failing to find a clear association between triglyceride levels and HTN [42]. This divergence may be due to lifestyle modifications or medication use among individuals with preexisting conditions (e.g., diabetes, cardiovascular disease, or HTN). Such factors could alter their lipid profiles [43, 44].

### Sex-specific predictors

In addition to common predictors, our study identified several sex-specific risk factors. In men, unintentional napping, recurrent headaches, and elevated triglyceride and cholesterol levels were significant predictors of HTN. These findings are in line with research linking sleep disturbances, such as frequent napping, to HTN through mechanisms involving poor sleep quality and metabolic dysregulation [45, 46]. While most studies associate long daytime naps (≥ 90 min) with HTN in women, a few suggest that napping may be a risk factor in men as well [47]. Moreover, previous studies have highlighted a connection between migraines and HTN, suggesting that individuals who experience migraines are at an increased risk of developing HTN [48, 49]. Recurrent headaches could be associated with higher blood pressure due to shared vascular or neurological mechanisms.

#### In women

Significant predictors of hypertension (HTN) included the use of sleeping pills, contraceptive drugs, and a history of kidney stones. Our analysis found a statistically significant association between contraceptive use and HTN, with a hazard ratio of 1.24; however, this effect size is modest and should be interpreted with caution. Previous research indicates that long-term use of sleeping medications may increase the risk of HTN and cardiovascular disease, potentially due to disruptions in circadian rhythms and heightened sympathetic nervous system activity [50, 51]. Similarly, oral contraceptives, particularly those containing estrogen, have been associated with increased blood pressure, especially in postmenopausal women. This study examined both low-dose (LD) and triphasic formulations, both of which contain estrogen and progesterone. Previous research has indicated that estrogen-only pills are more likely to raise blood pressure compared to progestin-only pills. Studies suggest that progestin-only pills either have no significant effect on blood pressure and cardiovascular disease or only slightly increase the risk. Consequently, it was challenging to isolate the effects of these hormonal pills when they were used together [52–54]. Our findings align with previous research demonstrating a relationship between kidney stones and hypertension (HTN) [55, 56]. While some studies have indicated that patients with hypertension are at an increased risk of developing urolithiasis, the design of this cohort study, focused on new hypertension cases, suggests that kidney stones may precede and increase the risk of hypertension. Additionally, shared pathophysiological mechanisms, such as impaired calcium metabolism, oxidative stress, and inflammation, may contribute to both conditions, warranting further investigation [57, 58].

### Behavioral factors: Smoking and opium use

The relationship between smoking, opium use, and hypertension (HTN) is complex, with conflicting findings reported across studies [18, 32, 48, 49, 59–66]. Some studies indicate that these behaviors may be associated with either an increased or decreased risk of HTN, while others find no significant relationship. For example, Najafipour et al. reported that occasional opium use is associated with a 42% increase in the risk of HTN, whereas continuous use is associated with a 36% increase [66]. In contrast, Rezaianzadeh et al. observed an inverse relationship between opium use and HTN [12]. Similarly, Kumar et al. identified an inverse relationship between smoking and HTN in men, while Gu et al. reported a higher incidence of HTN among female smokers [19]. Our findings indicate an association of smoking and opium use with an increased risk of HTN, particularly with longer durations of smoking. However, it is essential to note that this association does not imply causality and should be interpreted with caution. Several factors may explain the conflicting results in the literature. First, cross-sectional study designs may be limited by the potential for behavior modification following a diagnosis of hypertension (HTN). Furthermore, differences in the drugs studied across various research efforts may help explain the variability in findings [18, 48, 65].

### Health conditions and hypertension

Our study also confirmed a strong relationship between pre-hypertension and HTN in both sexes, a finding consistent with previous research [18, 67] Additionally, rheumatoid disease was identified as a significant predictor of HTN in both men and women, even after adjusting for medication use, including steroidal and non-steroidal anti-inflammatory drugs (NSAIDs), statins, and aspirin. This aligns with previous studies that suggest inflammation, oxidative stress, and long-term medication use may contribute to an increased prevalence of HTN in patients with rheumatoid disease [68–71]. Moreover, we found that individuals with a family history of HTN are at greater risk of developing the condition, with this risk being more pronounced in women than in men. Previous studies have similarly demonstrated that individuals with a family history of HTN are 2–4 times more likely to develop the condition compared to those without such a history [72–75]. This increased risk is attributed to genetic factors and differences in anthropometric parameters, such as waist circumference, BMI, and blood lipids, which are often higher in individuals with a family history of hypertension [74, 76]. A history of head injury with anesthesia was also associated with a higher risk of HTN in women. This finding is consistent with research by Izzy et al., who suggest that traumatic brain injury may have long-term effects on cardiovascular regulation, leading to a higher risk of chronic cardiovascular, endocrine, and neurological conditions [77–81].

### Iris color and hypertension

Our study found a lower risk of hypertension (HTN) among individuals with green irises, aligning with previous findings by Friedman et al. Although a definitive genetic link between eye color and blood pressure regulation has yet to be established, it is possible that certain genes influencing eye color may also play a role in blood pressure control. However, research specifically examining the relationship between iris color and blood pressure remains limited, indicating a need for more targeted studies in this area [82]. Conversely, the relationship between skin color and hypertension has been studied more extensively, though the findings have been inconsistent. While some studies report no significant association, others have observed a higher prevalence of hypertension among individuals with darker skin tones. These mixed findings underscore the complexity of the genetic and environmental factors involved in blood pressure regulation. Therefore, further research is necessary to clarify the potential mechanisms linking iris color to hypertension risk and to resolve these conflicting results [83, 84].

## Conclusion

This study highlights the importance of sex-specific analyses in understanding the incidence and predictors of hypertension (HTN). Our findings underscore the need for tailored approaches to HTN prevention and management that address the unique risk factors associated with each sex. Further research is essential to elucidate the mechanisms underlying these sex-specific differences in hypertension risk and to optimize HTN interventions for both men and women.

### Limitations and strengths

Our study has several notable strengths. First, as a prospective cohort study, it overcomes the temporal limitations inherent in cross-sectional studies, allowing for a clearer understanding of the relationship between predictive factors and the development of hypertension (HTN) over time. Second, we accounted for potential confounding factors by simultaneously analyzing multiple variables, enabling us to control for their effects and more accurately assess the independent associations between predictive factors and HTN. Data collection was conducted by experienced experts, with high blood pressure cases confirmed by two internists. This rigorous methodology enhances the reliability and validity of our results. Another strength of our study is its large sample size, which included participants from both rural and urban areas, as well as diverse cultural, social, and economic backgrounds. This diversity enhances the generalizability of our findings. However, we acknowledge that specific cultural, lifestyle (such as the consumption of herbal teas like thyme, which has been reported in other studies to reduce blood pressure [85]), genetic, and environmental factors unique to our study population may limit the applicability of these results to other populations. Therefore, while our results provide valuable insights, caution should be exercised when interpreting them across different regions. Despite these strengths, our study has certain limitations. One important consideration is the social stigma associated with drug and alcohol use in Iran, which may have led participants to underreport or conceal these behaviors, potentially introducing bias into the data related to these variables. Additionally, while we attempted to control for numerous potential confounders, unmeasured or residual confounding may still exist. These limitations should be considered when interpreting our findings.

## Data Availability

The data sets used and/or analyzed during the current study are available from the corresponding author upon reasonable request.n.baerade@yahoo.com.

## Abbreviations

HTN: Hypertension
CI: Confidence interval
WHR: Waist to Height Ratio
BMI: Body mass index
HDL: High-density lipoprotein
HR: Hazard rate
